# Cockroach protease allergen induces allergic airway inflammation via epithelial cell activation

**DOI:** 10.1038/srep42341

**Published:** 2017-02-15

**Authors:** Sagar L. Kale, Komal Agrawal, Shailendra Nath Gaur, Naveen Arora

**Affiliations:** 1 Allergy and Immunology Section, CSIR-Institute of Genomics and Integrative Biology, Delhi University Campus, Mall road, Delhi-110007, India; 2 Department of Respiratory Medicine, Vallabhbhai Patel Chest Institute, University of Delhi, Delhi, India

## Abstract

Protease allergens are known to enhance allergic inflammation but their exact role in initiation of allergic reactions at mucosal surfaces still remains elusive. This study was aimed at deciphering the role of serine protease activity of Per a 10, a major cockroach allergen in initiation of allergic inflammation at mucosal surfaces. We demonstrate that Per a 10 increases epithelial permeability by disruption of tight junction proteins, ZO-1 and occludin, and enhances the migration of Monocyte derived dendritic cell precursors towards epithelial layer as exhibited by trans-well studies. Per a 10 exposure also leads to secretion of IL-33, TSLP and intracellular Ca^2+^ dependent increase in ATP levels. Further, *in vivo* experiments revealed that Per a 10 administration in mice elevated allergic inflammatory parameters along with high levels of IL-33, TSLP, IL-1α and uric acid in the mice lungs. We next demonstrated that Per a 10 cleaves CD23 (low affinity IgE receptor) from the surface of PBMCs and purified B cells and CD25 (IL-2 receptor) from the surface of PBMCs and purified T cells in an activity dependent manner, which might favour Th2 responses. In conclusion, protease activity of Per a 10 plays a significant role in initiation of allergic airway inflammation at the mucosal surfaces.

The prevalence of allergic diseases is increasing worldwide from past few decades in developing as well as developed countries^1^. Allergic inflammatory responses are initiated on allergen uptake by APCs, predominantly DCs and subsequent polarisation of the T cell towards Th2 2. Though studies have tried to elucidate the protein characteristics that confer them with the ability to induce Th2 responses, it is still unclear why some proteins are allergenic while others are not^3^. Recent studies have demonstrated the role of biological properties like proteolytic activity (HDM, cockroaches, fungal extracts) and phospholipase activity (bee venom) of some allergens in development of Th2 immune responses^4^,^5^. Several allergens from a variety of sources viz., HDM, molds and cockroaches have proteolytic activity 6,7. These proteases have been shown to skew the immune response towards Th2 by the virtue of their proteolytic activity^8^,^9^.

Dendritic cells, the professional antigen presenting cells of the immune system are the sentinels of immunity and tolerance. Dendritic cells act as a bridge between innate immune sensing and initiation of adaptive immune responses^2^. Previously, Per a 10, a major serine protease allergen from American cockroaches (*Periplaneta americana*) has been shown to bias dendritic cell mediated polarisation of naïve T cells towards Th2. But the effect of Per a 10 exposure on the migration of DC precursors in the airway epithelium remains to be elucidated. Further, protease allergens are known to activate airway epithelial cells to secrete a wide array of chemokines and cytokines. Emerging studies have determined the role of epithelial cytokines, chemokines and mediators in orchestrating the local inflammatory responses and have directly implicated epithelial cells in Th2 responses^10^,^11^. Per a 10, has also been shown to activate airway epithelial cells to secrete IL-6, IL-8 and GMCSF in an activity dependent manner via PAR-2 receptors^12^. Airway epithelium acts as a first line of defence for the inhaled particles and forms a physical as well as a chemical barrier that shields immune cells from the external environment^13^. For sensitization, allergens need to cross the epithelial barrier to interact with subepithelial dendritic cells so that they are processed and presented^4^. This epithelial barrier is maintained by tight junctions that allow cell to cell contacts and defective barrier plays a role in pathogenesis of allergic diseases like asthma and dermatitis^13^. Proteases like Der p 1, Pen c 13 etc. disrupt airway epithelial barrier and contribute to sensitization and allergic responses^14^,^15^,^16^. Mucosal epithelial cells not only form a barrier for inhaled environment but are able to modulate innate and adaptive immune responses^17^.

Though proteolytic activity of Per a 10 causes allergic airway inflammation, its role in creating a Th2 microenvironment remains to be deciphered. In this study, we investigated the role of serine protease activity of Per a 10 in initiating and promoting allergic responses at mucosal surfaces.

## Results

### Per a 10 increases epithelial permeability in a time and activity dependent manner

**Figure 1:**
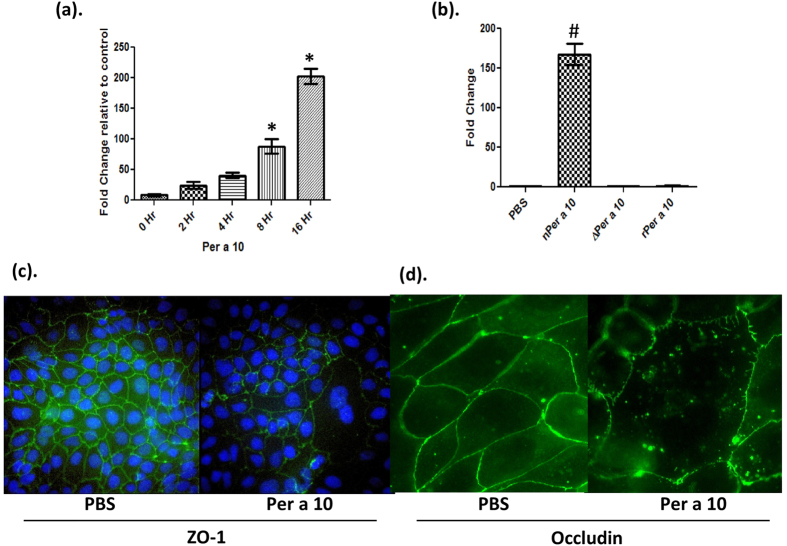
Per a 10 increases epithelial permeability. Calu-3 cells grown on apical surface of transwell plates at an air liquid interface were exposed to 10 μg of either active or inactive Per a 10 and RITC labelled dextran was added at the apical surface. 50 μl of media was collected from the basolateral and apical compartment at different time points and analyzed for RITC dextran by its absorbance at excitation/emission of 530/590 nm. (**a**) Fold change in transepithelial permeability with respect to PBS stimulation of Calu-3 cells when exposed to active Per a 10 for different time points (0 hr to 16 hr). (**b**) Fold Change in transepithelial permeability with respect to PBS stimulation when Calu-3 cells were exposed to 10 μg of active (n) Per a 10 or inactive Per a 10 (ΔPer a 10 and rPer a 10) for 16 hours. Active Per a 10 disrupts tight junction proteins. Calu-3 cells were grown till confluency on coverslips, exposed with Per a 10 for four hours. (**c**) Expression of ZO-1 and (**d**) Occludin as assessed by immunoflourescence. Data are representative of one of the three independent experiments performed. Data are presented as mean ± SEM for three independent experiments performed. *,#P < 0.05.

Per a 10 was isolated from cockroach crude extract and it resolved as a single band at 28 kDa on SDS-PAGE^12^. The endotoxin levels in Per a 10 were determined by LAL assay and were below the detection limit (0.01 ng/mg of protein) of the kit. Native Per a 10 was proteolytically active but both recombinant Per a 10 (rPer a 10) and heat inactivated Per a 10 (ΔPer a 10) were proteolytically inactive^12^. We investigated the effect of Per a 10 exposure on trans-epithelial permeability of Calu-3 cells, a human derived airway epithelial cell line cultured at air liquid interface, by using fluorescently labelled dextran. On exposure with native Per a 10 the trans-epithelial permeability of Calu-3 cells increased in a time dependent manner but significant increase in the permeability was observed only after 8 h of exposure (Fig. 1a). Next, to investigate the effect of serine protease activity of Per a 10 on epithelial permeability, Calu-3 cells grown at air liquid interface were exposed either to proteolytically active or inactive Per a 10. Both, rPer a 10 and ΔPer a 10 did not cause any significant change in the epithelial permeability as compared to the PBS exposed cells (Fig. 1b), demonstrating the role of protease activity in increased epithelial permeability.

Tight junctions are apical cell-cell contacts in polarised cells like epithelia and form a physical barrier^18^. The significant increase in permeability of Calu-3 cells caused by Per a 10 exposure may be due to cleavage of inter junctional complexes. So, we next sought to determine the effect of Per a 10 exposure on tight junctional proteins ZO-1 and Occludin of Calu-3 cells by immunofluorescence. As shown in the Fig. 1(c) ZO-1 and Occludin staining in the control PBS exposed Calu-3 cells was continuous at the whereas in Per a 10 treated cells it was discontinuous and disrupted with reduced fluorescence (Fig. 1c) depicting cleavage of these proteins by Per a 10.

### Per a 10 induces migration of dendritic cell precursors

**Figure 2:**
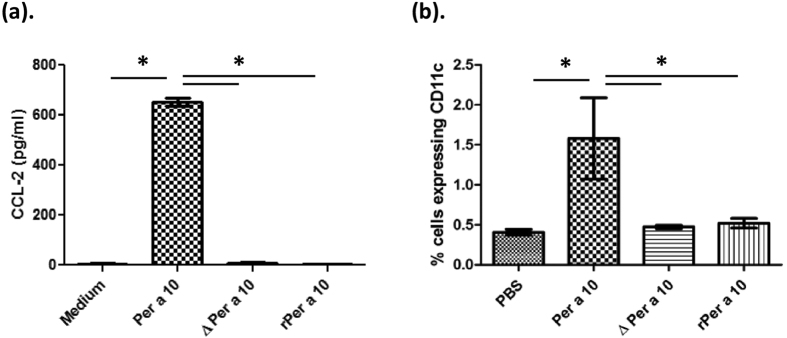
Per a 10 exposure leads to dendritic cell migration. Serum starved BEAS-2B cells were stimulated either with 10 μg of Per a 10, ΔPer a 10 or rPer a 10 for 24 hours and (**a**) CCL-2 levels in the supernatant assessed by ELISA. For migration studies, BEAS2B cells were cultured at the basal compartment of transwells, serum starved and stimulated with Per a 10. Dendritic cell precursors were generated from PBMCs of allergic patients and cultured in the upper chamber of the transwells. (**b**) Percent of DC precursors migrated from the upper chamber to the lower chamber of transwell plate containing BEAS-2B cells stimulated with active or inactive Per a 10. Data are presented as mean ± SEM for three independent experiments performed. * P < 0.05 as compared to Per a 10 stimulated cells.

CCL-2 levels are increased in bronchoalveolar lavage of asthmatic patients. Bronchial epithelial cells exposed to active Per a 10 showed increased secretion of CCL-2 in the supernatant as compared to inactive Per a 10 stimulated cells (Fig. 2a). PBMCs are the source for recruitment of DC precursors during allergen challenge; we isolated CD14+ monocytes from PBMCs, obtained from cockroach hypersensitive patients and cultured them in presence of rhIL-4 and rhGMCSF to generate DC precursors. We then checked the role of Per a 10 activated bronchial epithelial cells in migration of DC precursors by co-culture experiments. Per a 10 stimulated BEAS-2B cells caused migration of DCs from upper chamber to lower chamber of trans-wells as shown in Fig. 2b as compared to unstimulated or inactive Per a 10 stimulated epithelial cells.

### Per a 10 cleaves CD23, a low affinity IgE receptor

**Figure 3:**
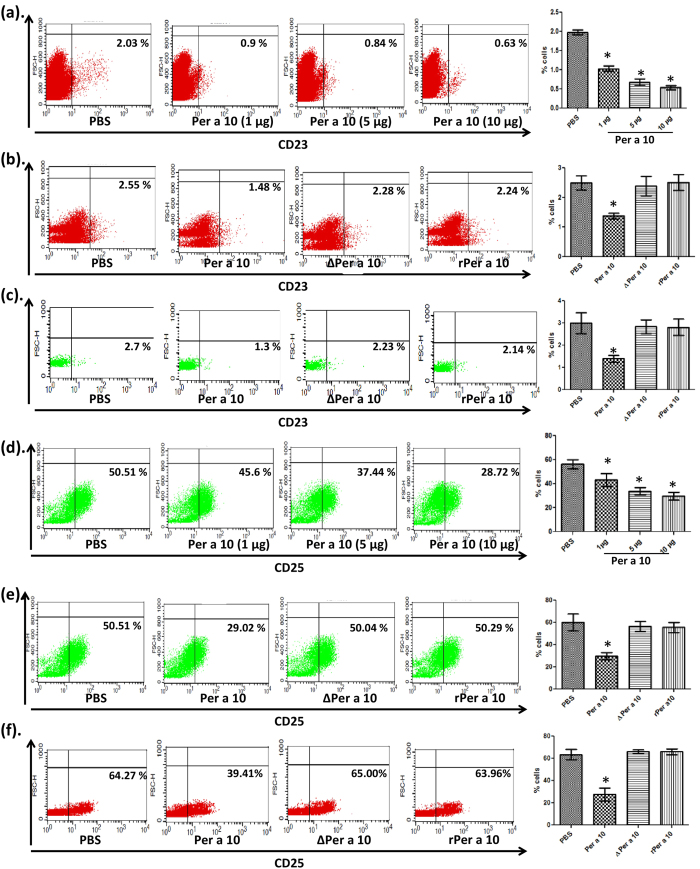
Per a 10 cleaves CD23, a low affinity IgE receptor and CD25, an IL-2 receptor subunit. PBMCs from normal healthy donors were cultured for 72 hours in the presence of PHA (5 μg/ml) in RPMI-1640 supplemented with 10% FBS. Cells were incubated with different stimuli for 4 hours, stained with FITC labelled anti CD23 or anti CD25 antibody and analysed by flow cytometry. B cells and CD4+ T cells were isolated from PBMCs using MACS, incubated with 10 μg of either active or inactive Per a 10 for 4 hrs and were stained with anti CD23 or CD25 antibody. (a) CD23 expression on PBMCs exposed with native active Per a 10 at varying concentrations, (**b**) CD23 expression on PBMCs exposed with 10 μg of Per a 10, ΔPer a 10 or rPer a 10. (**c**) Expression of CD23 on purified B cells exposed to 10 μg of Per a 10, ΔPer a 10 or rPer a 10, (**d**) CD25 expression on PBMCs exposed with Per a 10 at different concentrations, (**e**) CD25 expression on PBMCs exposed with 10 μg of Per a 10, ΔPer a 10 or rPer a 10, (**f** ) Expression of CD25 on purified CD4+ T cells. Dot plots are representative of one of the three independent experiments. Data presented as mean ± SEM of three independent experiments. *P < 0.05 as compared to PBS exposed cells.

CD23 is a regulator of IgE network. Binding of membrane bound CD23 (mCD 23) on B cells to IgE mediates a negative feedback loop for IgE synthesis whereas sCD23 mediates IgE synthesis promoting pathway^19^. We tried to elucidate the effect of protease activity of Per a 10 on membrane bound CD23. PBMCs were isolated and incubated with different concentrations of Per a 10 and analysed for surface expression of CD23 by flow cytometry. Percent of cells expressing CD23 decreased with increasing concentration of Per a 10 (Fig. 3a). There is approximately half fold reduction in cells expressing CD23 on treatment with native active Per a 10 as compared to the PBS and inactive Per a 10 exposed cells (Fig. 3b). Cells exposed to rPer a 10 and ΔPer a 10 showed no change in percent cells expressing CD23, as compared to PBS treated cells, suggesting role of proteolytic activity in reduced CD23 expression. Further, when purified B cells were exposed to Per a 10, percent cells expressing CD23 was significantly lower than ΔPer a 10 and rPer a 10 exposed cells (Fig. 3c).

### Per a 10 cleaves the α subunit of IL-2 receptor (CD25)

IL-2R is crucial for the propagation of Th1 cells and its cleavage can bias the immune response towards Th2 20. As Per a 10 cleaves CD23, we next checked for the cleavage of CD25 on PBMCs from healthy individuals that were exposed to native active Per a 10 or inactive ΔPer a 10 or rPer a 10 for 4 hrs. The surface expression of CD25 was analysed with flow cytometry. After exposure to Per a 10 the percentage of cells expressing CD25 reduced as compared to PBS stimulated cells in a dose dependent manner (Fig. 3d). There was no change in the percentage of cells expressing CD25 when exposed to inactive Per a 10 as compared to PBS stimulated cells (Fig. 3e) depicting activity dependent cleavage of CD25. Similarly, purified CD25+ CD4+ T cells exposed to native Per a 10 showed significant decrease in CD25 expression as compared to ΔPer a 10 and rPer a 10 treated cells (Fig. 3f).

### Per a 10 activity induces secretion of TSLP and IL-33 from bronchial epithelial cell line BEAS-2B

**Figure 4:**
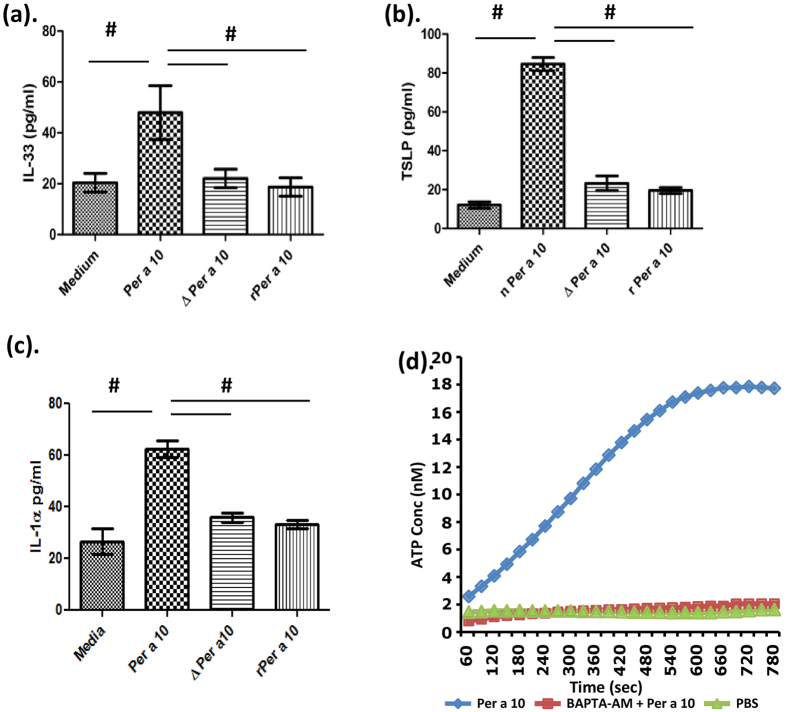
Per a 10 activates bronchial epithelial cells. Serum starved BEAS-2B cells were stimulated either with 10 μg of Per a 10, ΔPer a 10 or rPer a 10 for 24 hours and (**a**) IL-33 (**b**) TSLP and (**c**) IL-1α levels in the supernatant were assessed by ELISA. (**d**) ATP levels evaluated using luciferase/luciferin bioluminescence ATP determination kit (Invitrogen/Life technologies) and a luminometer, in supernatant of BEAS-2B cells grown in 96 well plate and stimulated with Per a 10 or PBS or cells incubated with BAPTA-AM and Per a 10. ^#^P < 0.05 as compared to Per a 10 stimulated cells.

Previously, we have shown that Per a 10 activates airway epithelial cells to secrete proinflammatory cytokines^12^. The effect of Per a 10 activity on induction of IL-33 and TSLP from bronchial epithelial cell line BEAS-2B was assessed in this study. Serum starved BEAS-2B cells were exposed to 10 μg of active or inactive Per a 10 and the supernatant was analyzed for the secretion of IL-33 and TSLP by ELISA. As shown in the Fig. 3, active Per a 10 induces significant increase in secretion of IL-33 (Fig. 4a) and TSLP (Fig. 4b) from BEAS-2B cells. There was no significant change in the levels of cytokines in the supernatants of inactive Per a 10 (ΔPer a 10 and rPer a 10) stimulated BEAS-2B cells (Fig. 4a,b).

### Per a 10 elevates ATP levels in BEAS-2B cells

Extracellular ATP, a danger alarmin has been previously shown to trigger the release of IL-33 and initiate Th2 type responses^21^. As Per a 10 elevates the secretion of IL-33 from exposed BEAS-2B cells, we checked if Per a 10 also increases ATP release from BEAS-2B cells. Per a 10 exposure increased ATP levels in the BEAS-2B cell supernatant in a time dependent manner (Fig. 4d). Next, to assess whether intracellular Ca^2+^ mobilisation is involved in ATP release, BEAS-2B cells pre-exposed to BAPTA-AM (an intracellular calcium chelator) were stimulated with Per a 10 and ATP release with time was monitored. As shown in Fig. 4d chelation of intracellular Ca^2+^ prevented the release of ATP in Per a 10 exposed BEAS-2B cells indicating its upstream role in ATP release. Also, it has been demonstrated that IL-1α triggers the release of IL-33. IL-1α levels as shown in Fig. 4c were significantly elevated in Per a 10 stimulated BEAS-2B cells as compared to inactive Per a 10 stimulated BEAS2B cells demonstrating the role of IL-1α in Per a 10 induced epithelial activation.

### Intranasal administration of Per a 10 induces allergic airway inflammation in mice

**Figure 5:**
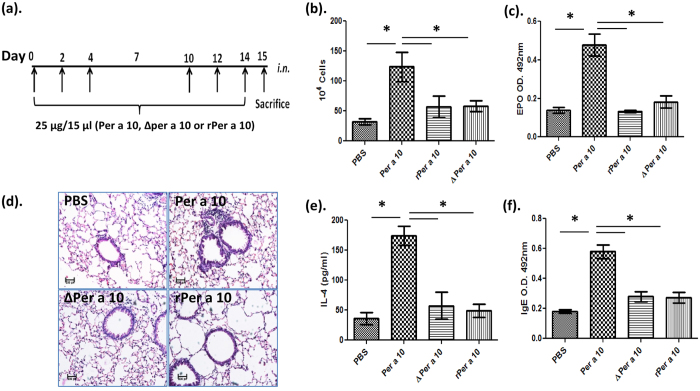
Active Per a 10 induces airway inflammation in Balb/c mice. Mice were exposed with active or inactive Per a 10 *i.n* three days a week for two weeks and were sacrificed on 15^th^ day. (**a**) The protocol for *i.n* sensitisation and challenge of mice with Per a 10, ΔPer a 10, rPer a 10 or PBS. (**b**) Total cell count and (**c**) EPO activity in BALF, (**d**) haematoxylin and eosin stained lung sections indicating cellular infiltration (**e**) IL-4 levels in the BALF and (**f** ) serum IgE levels. Data presented as mean ± SEM of 6 mice per group and are representative of one of the two independent experiments performed. *P < 0.05 as compared to active Per a 10 sensitized mice.

To determine the role of protease activity of Per a 10 on allergic airway inflammation, Balb/c mice were intranasally administered either with active or inactive Per a 10 (Fig. 5a) and allergic inflammatory parameters were analysed. Total cell count (Fig. 5b) and eosinophil peroxidise activity (EPO) (Fig. 5c) were higher in the BALF of Per a 10 immunised mice as compared to PBS administered mice and inactive Per a 10 (ΔPer a 10 and rPer a 10) immunised mice. There was no significant change in the total cell count and EPO activity between PBS administered and inactive Per a 10 administered mice (Fig. 5b,c). Further, haematoxylin and eosin staining of the lung sections revealed increment in cellular infiltration in mice administered with active Per a 10 as compared to mice administered either with inactive Per a 10 or PBS (Fig. 5d). Active Per a 10 administered mice also showed elevated levels of IL-4 (Fig. 5e) in BALF and IgE (Fig. 5f) in serum in comparison to the mice exposed to PBS or inactive Per a 10.

### Active Per a 10 elevates IL-33 and TSLP levels in the mice lungs

**Figure 6:**
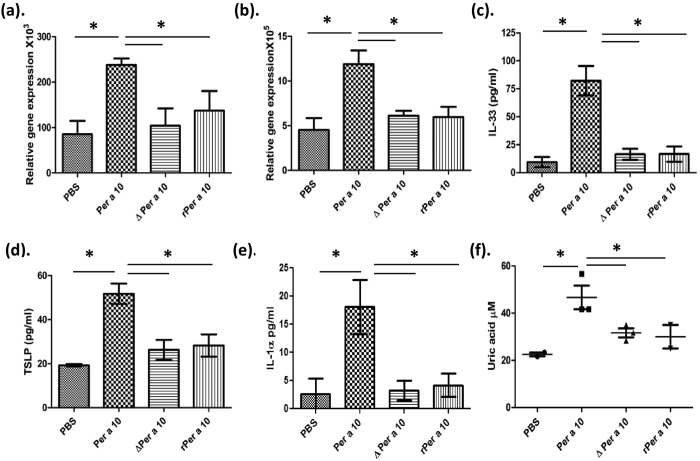
Per a 10 elevates IL-33, TSLP, IL-1α and uric acid levels in mice lungs. Mice were sensitized intranasally with either active or inactive Per a 10 for three days a week for two weeks and euthanized on 15^th^ day. BALF was collected: total RNA was isolated from the left lobe of the lung and expression of TSLP and IL-33 mRNA was assessed by quantitative realtime PCR. mRNA levels of (**a**) IL-33 and (**b**) TSLP in the lungs; (**c**) IL-33 in lung homogenate (**d**) TSLP (**e**) IL-1α and (**f**) Uric acid levels in the BALF of PBS, active Per a 10 and inactive Per a 10 sensitized mice. Data presented as mean ± SEM of 6 mice per group and are representative of one of the two independent experiments performed. *P < 0.05 as compared to active Per a 10 sensitized mice.

As Per a 10 induced secretion of IL-33 and TSLP from BEAS-2B cells, we checked whether Per a 10 exposure leads to any changes in the levels of these cytokines in mice model of allergic airway inflammation. Quantitative PCR analysis of IL-33 and TSLP mRNA expression in mice sensitized with active Per a 10 and inactive Per a 10 (ΔPer a 10 and rPer a 10) revealed that the expression of IL-33 (Fig. 6a) and TSLP (Fig. 6b) is increased in active Per a 10 sensitized mice as compared to inactive Per a 10 sensitized mice.

Next we sought to determine the effect of Per a 10 sensitization on IL-33 and TSLP levels in the lung homogenate and BALF of mice sensitized with either active or inactive Per a 10. Levels of both the cytokines were elevated in the lungs of active Per a 10 sensitized mice as compared to PBS sensitized mice (Fig. 6c,d). There was no significant difference in the levels of both these cytokines in inactive Per a 10 sensitized mice as compared to PBS sensitized mice (Fig. 6c,d).

### Per a 10 induces IL-1α secretion and uric acid release upon administration in the lungs

In corroboration with our *in vitro* results active Per a 10 exposure led to higher levels of IL-1α in the BALF as compared to inactive Per a 10 exposed mice (Fig. 6e). Uric acid is a danger associated molecule and precedes IL-33 secretion. The levels of uric acid in BALF of active Per a 10 sensitized mice were higher than in the inactive Per a 10 sensitized mice (Fig. 6f). Further, there was no difference in the levels of uric acid in inactive Per a 10 sensitized mice as compared to PBS sensitized control group (Fig. 6f). This indicates that respiratory mucosa on encountering protease allergen like Per a 10 releases uric acid in the airway lumen that can further lead to increased levels of IL-33 and TSLP, thus initiating allergen specific Th2 responses.

## Discussion

Prevalence of allergic airway diseases is on the rise in past few decades, yet the mechanisms that lead to Th2 immune responses are not fully understood. Exogenous proteases from house dust mites, cockroaches, fungi and pollen or endogenous proteases viz. neutrophil elastase, mast cell tryptase etc. are implicated in initiating and exacerbating allergic responses^22^,^23^,^24^,^25^. Airway epithelial cells act as a first line of defence for inhaled pathogens and aeroallergens^26^. Lung epithelial cells form a tight, virtually impermeable barrier for macromolecules and regulates paracellular transport of inhaled material by the formation of tight junctions at the apical surfaces^18^. In order to activate the immune cascade, allergens need to disrupt the tight epithelial barrier and gain access to the immune components of the host. Our study demonstrates that Per a 10 increases trans-epithelial permeability of Calu-3 cells by disruption of tight junctions which is in accordance with previous studies carried out with Der p 1, a cysteine protease and pollen proteases^15^,^27^. Also, impaired epithelium is evident in the lungs of allergic asthmatics^28^, suggesting possible damage by the inhaled allergens. Previously, Per a 10 has been shown to provide adjuvant effect to other bystander allergens in the same environment^8^. Per a 10, by compromising the epithelial barrier may facilitate the entry of other allergens across epithelial barrier and thus can act as an adjuvant to other bystander allergens in the same environment as reported earlier^8^.

Allergens favour Th2 polarisation of naive T cells, but the mechanism of this polarisation is still not clearly understood. Our results demonstrate that Per a 10 cleaves CD25 in an activity and dose dependent manner. CD25 is a 55 kDa subunit of IL-2 receptor^20^. IL-2 plays an important role in T cell proliferation and IL-2 receptor is pivotal for propagation of Th1 cells^20^. Thus CD25 cleavage by Per a 10 can hamper Th1 development, favouring Th2 propagation^29^. Previously, Der p 1 along with endogenous proteases like elastases and MMP-9 have been shown to cleave CD25 30,31,32. Th2 cells secrete myriad of Th2 cytokines, of which IL-4 induces IgE class switching in B cells, also patients with atopic diseases show elevated IgE levels^33^. CD23 a low affinity IgE receptor (FcεRII) exists in membrane bound (mCD23) and soluble form (sCD23) and is a key regulator of IgE synthesis. Both mCD23 and sCD23 differentially regulate IgE. Free IgE binds to membrane bound CD23 and down regulates its own production through negative feedback loop^34^. sCD23 binds to IgE, restricting its binding to membrane bound CD23 thus interrupting this feedback mechanism thereby promoting further IgE production. Endogenous proteases like disintegrin/metalloproteinase family members along with exogenous protease allergens (Der p 1) have been implicated in CD23 cleavage^35^,^32^. Per a 10 cleaves CD23 and can disrupt the IgE regulation resulting in its increased production. This also explains elevated systemic and local IgE levels in mice sensitized and challenged with Per a 10 as observed in the present as well as other studies^8^,^36^. Soluble CD23 levels are elevated in a variety of diseases including rheumatoid arthritis (joints and synovial fluids), Sjögren’s syndrome (plasma and saliva) and in systemic lupus erythematosus (systemic) patients^37^,^38^,^39^. CD23 is a potential target for therapeutic intervention as it is involved in immune and IgE regulation that makes CD23 cleavage as an active area of research^40^.

Further, our results demonstrate that bronchial epithelial cell activation by Per a 10 induces migration of dendritic cell precursors towards the epithelial layer. Dendritic cells are the sentinels of immune system and play a critical role in initiation and maintenance of allergic Th2 responses^41^,^42^. Immature dendritic cells form a dense network below the epithelial cells in the lungs and sense the environment for inhaled particles in conjunction with epithelial cells^43^. There exists an extensive crosstalk between epithelial cells and dendritic cells, which can influence the functioning of dendritic cells^44^. Reports suggest that there is an increase in number of dendritic cells in the lungs of allergic asthmatic patients as compared to normal healthy individuals which increases further upon allergen challenge and that epithelial cells play a role in dendritic cell precursor migration to the lung epithelial surfaces^45^.

To assess the role of protease activity and epithelial cytokines in development of Th2 responses, we generated an intranasal Per a 10 sensitized and challenged mice model of allergic airway inflammation. Mice sensitized and challenged with proteolytically active Per a 10 developed allergic airway inflammation symptoms. Epithelial activation is associated with allergen sensitization and is a characteristic of allergic asthma and rhinitis^26^. Emerging reports have implicated epithelial cytokines IL-33 and TSLP in allergic reactions^46^,^47^,^48^. Our results indicate that Per a 10 activates airway epithelial cells to elevate IL-33 and TSLP levels. We next corroborated our results obtained in mice model of allergic airway inflammation in an *in vitro* model by exposing BEAS-2B cells with active and inactive Per a 10. BEAS-2B cells stimulated with active Per a 10 induced secretion of IL-33 and TSLP. IL-33 is a member of IL-1 family of cytokines and acts via its receptor ST2 that is expressed by a variety of cells like eosinophils, mast cells, basophils and natural killer cells^49^. It is a constitutively expressed cytokine, usually found sequestered in the nucleus^50^ and is secreted upon epithelial cell activation. Proteases from *Alternaria alternata* and HDM are reported to induce release of IL-33 51,52. Activation of IL-33/ST2 pathway triggers the release of proinflammatory cytokines, chemokines and mediators, induces systemic Th2 responses and contributes to allergen induced airway inflammation and hyper responsiveness^53^,^54^. TSLP is another epithelial cytokine implicated in pathogenesis of allergic diseases that directs DCs towards Th2 responses and links epithelial cell activation to dendritic cell mediated immune regulation^55^. TSLP expression is increased in bronchial mucosa of severe asthmatics^56^. TSLP induces OX40L expression on DCs, which is required for polarisation of naïve CD4+ T cells towards Th2 57. Previously, Per a 10 has been shown to induce OX40L expression on DCs^36^ suggesting an important role of TSLP-DC-OX40L axis in initiating and maintaining Th2 responses.

IL-1α, uric acid and ATP have been implicated in promoting allergic Th2 responses^4^. We found elevated levels of uric acid and IL-1α in the BALF of active Per a 10 administered mice as compared to inactive Per a 10 and PBS administered mice. IL-1α levels were also elevated in the supernatant of active Per a 10 exposed BEAS-2B as compared to media and inactive Per a 10 stimulated cells. IL-1α plays an important role in Th2 sensitization to HDM. IL-1α was shown to act upstream of cytokine cascade that leads to activation of epithelial and dendritic cells in response to HDM sensitization^4^. Uric acid, a by-product of metabolism, is an alarmin that is secreted by stressed cells^58^. Intraperitoneal administration of OVA along with uric acid crystals led to Th2 immune responses like increased eosinophilia, goblet hyperplasia, along with induction of OVA specific IgE, IgG1 and IgG2a 59. Uric acid plays a critical role in peanut sensitization as peanut sensitized mice showed altered purine metabolism with increased serum uric acid levels which were also found to be elevated in peanut allergic children^60^. Cysteine proteases like bromelin and papain have been shown to induce release of uric acid in the airway lumen^61^. Further, administration of uric acid in the naive mice has been shown to induce IL-33 release and Th2 responses^59^. Exposure of bronchial epithelial cells to Per a 10 leads to increased secretion of ATP. ATP is another danger signal that is elevated in BALF of asthmatics and mice models of allergic asthma^62^. Previously, we have demonstrated the pivotal role protease activity of Per a 10 plays in the activation of airway epithelial cells and subsequent secretion of pro-inflammatory cytokines^12^. Inactive Per a 10 did not show any change in the levels of TSLP, IL-33 and IL1α, which were elevated after Per a 10 stimulation. The secretion of these cytokines seems to be dependent on the protease activity of Per a 10, the exact mechanism of which remains to be deciphered. It has been reported that *Alternaria alternata* exposure leads to elevated ATP levels which in turn results in IL-33 release and is dependent on protease activity^51^. The *Alternaria alternata* induced IL-33 secretion is mediated by ATP via activation of purigenic receptors^21^. Also, the activation of PAR-2 receptors and Calcium ion mobilization were shown to be key features associated with Per a 10 induced epithelial cell activation^12^. Jairaman et al., evaluated the underlying mechanism of Ca^2+^ mobilization after stimulation with cockroach and house dust mite extract and demonstrated that cockroach extract mediates Ca^2+^ mobilization via PAR-2 receptors and is independent of Ca^2+^ mobilization induced by purinergic receptor stimulation (P2Y) by UTP 63. In the present study ATP released after Per a 10 stimulation was dependent on intracellular Ca^2+^ mobilisation but the exact pathway responsible for ATP secretion after protease allergen stimulation is still unknown. Based on the results of our study and relevant literature it is now known that there exists a complex interplay between PAR-2 receptors, Ca^2+^ mobilization, ATP secretion and purinergic receptors in the regulation of various cytokines leading to Th2 responses. A detailed mechanistic study taking into consideration the above mentioned factors will be helpful in unravelling the signalling mechanisms employed by protease allergens in activating airway epithelial cells.

In conclusion, our results demonstrate that Per a 10 enhances epithelial permeability, activates airway epithelial cells, enhances IgE production and induces Th2 responses by the virtue of its proteolytic activity. Our study indicates that apart from genetic predisposition, intrinsic biochemical activity of certain allergens (protease activity) plays a critical role in initiating and promoting allergic airway responses at mucosal surfaces.

## Methods

### Isolation of Per a 10

Per a 10 was isolated from whole body cockroach extract by affinity chromatography as described elsewhere^64^. Per a 10 was cloned using gene specific primers, expressed in *E.coli* and purified using Ni-NTA affinity chromatography^65^. Per a 10 was boiled at 90 °C for 90 minutes to inactivate its protease activity. The protease activity of Per a 10, ΔPer a 10 and rPer a 10 was determined quantitatively by azocollagen assay 12. To assess endotoxin levels in the protein LAL chromogenic endotoxin quantitation kit was used using manufacturer’s instructions^36^.

### Permeability Assay

Calu-3 cells were cultured at air liquid interface in EMEM supplemented with 10% FBS, 100 U/ml penicillin and 100 ug/ml streptomycin at 37 °C in 5% CO_2_ to analyze the changes in transepithelial permeability^15^. Briefly, the cells were cultured in a 24 well transwell plate till formation of monolayer and incubated overnight in serum free EMEM. Cells were stimulated with 10 μg of Per a 10, ΔPer a 10 or rPer a 10. RITC labeled dextran 70 S (50 μl, 100 μM) was added at the apical surface of transwell plates. For changes in transepithelial permeability 50 μl of media was collected from the basolateral compartment at different time points and its absorbance at excitation/emission of 530/590 nm was measured. Change in permeability was calculated as fold change with respect to PBS stimulated cells.

### Immunofluorscence staining of junctional proteins

Calu-3 cells grown on coverslips till confluency were incubated in serum free media for 8 hours and exposed to either Per a 10 or PBS for 16 hours. The coverslips were removed and cells fixed in cold methanol for 10 mins, blocked with 3% (w/v) BSA in PBS. The cells were permeabilized with 0.3% triton X 100 for 10 mins at 4 °C and blocked with BSA (10% w/v in PBS) for 1 hour at RT. Cells were stained with mouse monoclonal anti-occludin (Life Technologies, Carlsbad, CA, USA) or rabbit polyclonal anti ZO-1 antibody (Life Technologies, Carlsbad, CA, USA) diluted (1:200) in 3% BSA (w/v in PBS) for 1 hour at RT washed with PBS 3 times and incubated for 2 hrs with Alexaflour 488 labelled secondary antibody (Life Technologies, Carlsbad, CA, USA). These cover slips were mounted on slides and analyzed using fluorescent microscopy (Leica DMI 6000).

### Monocyte isolation, generation of MDDCs and co-culture experiments

Monocytes were isolated from the PBMCs of cockroach hypersensitive patients, and MDDCs generated as described previously^65^. The monocytes cultured for five days in these cytokines were characterised for the expression of CD11c and was used in migration studies.

For transwell migration assays BEAS-2B cells were cultured in the lower chamber of 24 well transwell plate till 70–80% confluency. The cells were starved in epithelial cell basal media overnight exposed to 10 μg of active Per a 10 and the MDDC precursors (1 × 10^6^) were added to the upper chamber and incubated for 24 hours in serum free RPMI. After dissociating the BEAS-2B cells with a cell dissociating medium the cells were stained with PE labelled anti-human CD11c antibody or an isotype control and acquired using BD FACS calibur and analyzed by Cell Quest Pro.

### B cell and CD4+ T cell isolation

B cells and CD4+ T cells were purified from PBMCs by using MACS B-CLL isolation kit and human CD4+ isolation kit II (Miltenyi Biotec, Gladbach, Germany) following manufacturers instruction.

### Stimulation of cells and staining for flow cytometry

1 × 10^6^ Cells (PBMCs, B cells or CD4+ T cells) were incubated with Per a 10, rPer a 10 and ΔPer a 10 for 4 hours. Cells were washed with FACS buffer. The cells were stained with FITC labelled anti-CD23 (Life Technologies, Carlsbad, CA, USA) or anti-CD25 (Millipore, Massachusetts, USA) antibody for 90 minutes. The cells were then washed and analysed by flow cytometry for surface expression of CD23 and CD25 using BD FACS Calibur and analysed with cell quest pro.

### Cell culture and stimulation with Per a 10

BEAS-2B cells were cultured in BEGM (Invitrogen) till confluency at 37 °C and 5% CO_2_. Cells were then incubated in BEBM (without growth factors) overnight and exposed to 10 μg of active (Per a 10) or inactive (Δ Per a 10 and rPer a 10) Per a 10. Supernatant was collected and the levels of IL-33 and TSLP were measured by ELISA.

### ATP assays

ATP release was assayed by using a luciferase/luciferin bioluminescence ATP determination kit (Invitrogen/Life technologies) and a luminometer. Cells were grown in white 96 well plates till confluency. The cells were exposed to 2 μg Per a 10 and bioluminescence was measured in real time as per the manufacturer’s instructions. For time dependent ATP release assay bioluminescence was measured after every 30 seconds. The concentration of ATP was calculated from a standard graph prepared using known ATP concentrations.

### Animal experiment

Female Balb/c mice 6–8 week old were procured from National Institute of Nutrition (NIN), Hyderabad, India and were housed and acclimatize as described elsewhere. The study protocol was approved by animal ethics committee of Institute of Genomics and Integrative Biology, Delhi. All the experiments were performed in accordance with the guidelines of committee for the purpose of control and supervision of experiments on animals (CPCSEA), Ministry of Environment, Forests and Climate change, Government of India. Mice were divided randomly into 4 groups with each group containing 6 mice. The mice were anaesthetized with 3% isoflurane and allowed to inhale 25 μg (15 μl) of the respective allergen applied to snares with a pipette. Mice were administered with Per a 10, ΔPer a 10 or rPer a 10 on 0, 2^nd^, 4^th^, 10^th^, 12^th^ and 14^th^ day and were euthanized by *i.p.* injection of thiopentone (100 mg/kg) on 15^th^ day. PBS administered group of mice served as a negative control group. BALF (for ELISA, EPO and cell count), blood (for IgE) and lungs (for lung homogenate and histopathology) were collected and processed as described elsewhere^7^,^36^.

### Measurement of Per a 10 specific IgE in the sera of mice

Relative levels of Per a 10 specific IgE were measured in the serum samples of mice by indirect ELISA. Briefly, microtitre plates were coated with 100 ng of Per a 10/well in 100 μl of 0.1 M carbonate buffer (pH 9.6) and incubated overnight at 4 °C. After washing with PBS the plates were blocked with 3% defatted milk for 3 h at 37 °C. Sera samples were diluted 1:10 in PBS and 100 μl of the diluted sera was added in triplicates. The plates were incubated at 4 °C overnight. After washing with PBST (0.05% Tween-20 in PBS) to remove the unbound antibodies the plate was incubated with biotinylated anti-mouse IgE (2 μg/ml) at 25 °C for 90 min followed by streptavidin-peroxidase (1:1000) for 30 min. The plate was washed and was developed using o-phenylenediamine and absorbance was read at 492 nm 7.

### Quantitative PCR

Total RNA was isolated from lung tissue by homogenizing in 2 ml of Trizol (Life technologies) and quantified using nanodrop. cDNA preparation and quantitative PCR was carried out as previously described^36^. The primers used were as follows: IL-33: forward 5′GCTGCGTCTGTTGACACATT-3′ and reverse 5′CACCTGGTCTTGCTCTTGGT-3′; TSLP: forward 5′CGGATGGGGCTAACTTACA-3′ and reverse 5′TCCTCGATTTGCTCGAACTT-3′; β-actin: forward 5′-CGGTTCCGATGCCCGAGGCTCTT-3′ and reverse 5′-CGTCACACTTCATGATGGAATTGA-3′.

### Uric acid determination

The concentration of uric acid in the BALF of mice was determined by Amplex red uric acid/uricase kit. Briefly, 50 μl of samples were pipetted out in a 96 well plate and a 50 μl of working solution of Amplex Red reagent containing 0.4 U/ml HRP and 0.4 U/ml uricase was added to them. A standard curve ranging from 0–100 μl was prepared from the stock of 5 mM uric acid in 1X reaction buffer. The plate was incubated at 37 °C in dark for 30 minutes and absorbance was measured at 560 nm using a Benchmark plus microplate reader.

### ELISA

The concentrations of cytokines hIL-33, hIL-1α, hCCL-2 (R&D systems), hTSLP, mIL-33, mTSLP, mIL-4 and mIL-1α (eBiosciences) in the cell supernatant, Lung homogenate and in the BALF were estimated using paired antibodies following manufacturer’s instructions.

### Statistical analysis

Statistical analysis of results was done by using GraphPad Prism software (GraphPad Software, San Diego, CA, USA). The statistically significant difference was determined using one way ANOVA and p value < 0.05 was considered as statistically significant.

## Additional Information

**How to cite this article**: Kale, S. L. *et al*. Cockroach protease allergen induces allergic airway inflammation via epithelial cell activation. *Sci. Rep.* **7**, 42341; doi: 10.1038/srep42341 (2017).

**Publisher's note:** Springer Nature remains neutral with regard to jurisdictional claims in published maps and institutional affiliations.

## References

[b1] Pawankar R. Allergic diseases and asthma: a global public health concern and a call to action. World Allergy Organ. J. 7, 12 (2014). 24940476 10.1186/1939-4551-7-12 PMC4045871

[b2] Larché M., Akdis C. A. & Valenta R. Immunological mechanisms of allergen-specific immunotherapy. Nat. Rev. Immunol. 6, 761–71 (2006). 16998509 10.1038/nri1934

[b3] Huby R. D., Dearman R. J. & Kimber I. Why are some proteins allergens? Toxicol. Sci. 55, 235–246 (2000). 10828254 10.1093/toxsci/55.2.235

[b4] Willart M. a. M. . Interleukin-1 controls allergic sensitization to inhaled house dust mite via the epithelial release of GM-CSF and IL-33. J. Exp. Med. 209, 1505–1517 (2012). 22802353 10.1084/jem.20112691 PMC3409497

[b5] Dudler T. . A link between catalytic activity, IgE-independent mast cell activation, and allergenicity of bee venom phospholipase A2. J. Immunol. 155, 2605–13 (1995). 7544378

[b6] Chapman M. D., Wünschmann S. & Pomés A. Proteases as Th2 adjuvants. Current Allergy and Asthma Reports 7, 363–367 (2007). 17697645 10.1007/s11882-007-0055-6

[b7] Saw S., Kale S. L. & Arora N. Serine protease inhibitor attenuates ovalbumin induced inflammation in mouse model of allergic airway disease. PLoS One 7, (2012). 10.1371/journal.pone.0041107 PMC3400607 22829914

[b8] Sudha V. T., Arora N. & Singh B. P. Serine protease activity of per a 10 augments allergen-induced airway inflammation in a mouse model. Eur. J. Clin. Invest. 39, 507–516 (2009). 19397689 10.1111/j.1365-2362.2009.02112.x

[b9] Tripathi P., Kukreja N., Singh B. P. & Arora N. Serine protease activity of cur l 1 from curvularia lunata augments Th2 response in mice. J. Clin. Immunol. 29, 292–302 (2009). 19020963 10.1007/s10875-008-9261-9

[b10] Bartemes K. R. & Kita H. Dynamic role of epithelium-derived cytokines in asthma. Clinical Immunology 143, 222–235 (2012). 22534317 10.1016/j.clim.2012.03.001 PMC3358585

[b11] Bulek K., Swaidani S., Aronica M. & Li X. Epithelium: the interplay between innate and Th2 immunity. Immunol. Cell Biol. 88, 257–68 (2010). 20065993 10.1038/icb.2009.113

[b12] Kale S. L. & Arora N. Per a 10 activates human derived epithelial cell line in a protease dependent manner via PAR-2. Immunobiology 220, 525–32 (2015). 25468564 10.1016/j.imbio.2014.10.018

[b13] Holgate S. T., Roberts G., Arshad H. S., Howarth P. H. & Davies D. E. The role of the airway epithelium and its interaction with environmental factors in asthma pathogenesis. Proc. Am. Thorac. Soc. 6, 655–9 (2009). 20008870 10.1513/pats.200907-072DP

[b14] Chen J. C. . The protease allergen Pen c 13 induces allergic airway inflammation and changes in epithelial barrier integrity and function in a murine model. J. Biol. Chem. 286, 26667–26679 (2011). 21613216 10.1074/jbc.M110.193987 PMC3143630

[b15] Vinhas R. . Pollen proteases compromise the airway epithelial barrier through degradation of transmembrane adhesion proteins and lung bioactive peptides. Allergy Eur. J. Allergy Clin. Immunol. 66, 1088–1098 (2011). 10.1111/j.1398-9995.2011.02598.x 21480927

[b16] Wan H. . Quantitative structural and biochemical analyses of tight junction dynamics following exposure of epithelial cells to house dust mite allergen Der p 1. Clin Exp Allergy 30, 685–698 (2000). 10792361 10.1046/j.1365-2222.2000.00820.x

[b17] Kale S. & Arora N. Airway epithelial cells: Barrier and much more. Indian J. Allergy, Asthma Immunol. 27, 95 (2013).

[b18] Anderson J. M. & Van Itallie C. M. Physiology and function of the tight junction. Cold Spring Harbor perspectives in biology 1, (2009). 10.1101/cshperspect.a002584 PMC2742087 20066090

[b19] Cooper A. M. . Soluble CD23 controls IgE synthesis and homeostasis in human B cells. J. Immunol. 188, 3199–207 (2012). 22393152 10.4049/jimmunol.1102689 PMC3378639

[b20] Schulz O., Sewell H. F. & Shakib F. Proteolytic cleavage of CD25, the alpha subunit of the human T cell interleukin 2 receptor, by Der p 1, a major mite allergen with cysteine protease activity. J. Exp. Med. 187, 271–5 (1998). 9432986 10.1084/jem.187.2.271 PMC2212095

[b21] Kouzaki H., Iijima K., Kobayashi T., O’Grady S. M. & Kita H. The danger signal, extracellular ATP, is a sensor for an airborne allergen and triggers IL-33 release and innate Th2-type responses. J. Immunol. 186, 4375–87 (2011). 21357533 10.4049/jimmunol.1003020 PMC3062674

[b22] Asokananthan N. . House dust mite allergens induce proinflammatory cytokines from respiratory epithelial cells: the cysteine protease allergen, Der p 1, activates protease-activated receptor (PAR)-2 and inactivates PAR-1. J Immunol 169, 4572–4578 (2002). 12370395 10.4049/jimmunol.169.8.4572

[b23] King C., Brennan S., Thompson P. J. & Stewart G. a. Dust mite proteolytic allergens induce cytokine release from cultured airway epithelium. J. Immunol. 161, 3645–3651 (1998). 9759888

[b24] Miike S. & Kita H. Human eosinophils are activated by cysteine proteases and release inflammatory mediators. J. Allergy Clin. Immunol. 111, 704–713 (2003). 12704347 10.1067/mai.2003.1332

[b25] Reed C. E. & Kita H. The role of protease activation of inflammation in allergic respiratory diseases. Journal of Allergy and Clinical Immunology 114, 997–1008 (2004). 15536399 10.1016/j.jaci.2004.07.060

[b26] Wang Y., Bai C., Li K., Adler K. B. & Wang X. Role of airway epithelial cells in development of asthma and allergic rhinitis. Respir. Med. 102, 949–55 (2008). 18339528 10.1016/j.rmed.2008.01.017

[b27] Wan H. .. Der p 1 facilitates transepithelial allergen delivery by disruption of tight junctions. J. Clin. Invest. 104, 123–133 (1999). 10393706 10.1172/JCI5844 PMC408401

[b28] Swindle E. J., Collins J. E. & Davies D. E. Breakdown in epithelial barrier function in patients with asthma: identification of novel therapeutic approaches. J. Allergy Clin. Immunol. 124, 23–26 (2009). 19560576 10.1016/j.jaci.2009.05.037

[b29] Abbas A. K., Murphy K. M. & Sher A. Functional diversity of helper T lymphocytes. Nature 383, 787–793 (1996). 8893001 10.1038/383787a0

[b30] Sheu B. C. . A novel role of metalloproteinase in cancer-mediated immunosuppression. Cancer Res. 61, 237–42 (2001). 11196168

[b31] Bank U. . Selective Proteolytic Cleavage of IL-2 Receptor and IL-6 Receptor Ligand Binding Chains by Neutrophil-Derived Serine Proteases at Foci of Inflammation. J. Interf. Cytokine Res. 19, 1277–1287 (1999). 10.1089/107999099312957 10574621

[b32] Shakib F., Schulz O. & Sewell H. A mite subversive: cleavage of CD23 and CD25 by Der p 1 enhances allergenicity. Immunol. Today 19, 313–6 (1998). 9666604 10.1016/s0167-5699(98)01284-5

[b33] Gould H. J. & Sutton B. J. IgE in allergy and asthma today. Nat. Rev. Immunol. 8, 205–217 (2008). 18301424 10.1038/nri2273

[b34] Riffo-Vasquez Y. . The role of CD23 on allergen-induced IgE levels, pulmonary eosinophilia and bronchial hyperresponsiveness in mice. Clin. Exp. Allergy 30, 728–38 (2000). 10792366 10.1046/j.1365-2222.2000.00806.x

[b35] Fourie A. M., Coles F., Moreno V. & Karlsson L. Catalytic Activity of ADAM8, ADAM15, and MDC-L (ADAM28) on Synthetic Peptide Substrates and in Ectodomain Cleavage of CD23. J. Biol. Chem. 278, 30469–30477 (2003). 12777399 10.1074/jbc.M213157200

[b36] Agrawal K., Kale S. L. & Arora N. Protease activity of Per a 10 potentiates Th2 polarization by increasing IL-23 and OX40L. Eur. J. Immunol. 45, 3375–85 (2015). 26417883 10.1002/eji.201545734

[b37] Bansal A. . Soluble CD23 levels are elevated in the serum of patients with primary Sjögren’s syndrome and systemic lupus erythematosus. Clin. Exp. Immunol. 89, 452–5 (1992). 1387597 10.1111/j.1365-2249.1992.tb06979.x PMC1554466

[b38] Takei M. . Increased soluble CD23 molecules in serum/saliva and correlation with the stage of sialoectasis in patients with primary Sjögren’s syndrome. Clin. Exp. Rheumatol. 13, 711–5 (1995). 8835243

[b39] Huissoon A. P., Emery P., Bacon P. A., Gordon J. & Salmon M. Increased expression of CD23 in rheumatoid synovitis. Scand. J. Rheumatol. 29, 154–9 (2000). 10898066 10.1080/030097400750002012

[b40] Acharya M. . CD23/FcεRII: molecular multi-tasking. Clin. Exp. Immunol. 162, 12–23 (2010). 20831712 10.1111/j.1365-2249.2010.04210.x PMC2990925

[b41] Stockwin L. H., McGonagle D., Martin I. G. & Blair G. E. Dendritic cells: Immunological sentinels with a central role in health and disease. Immunol. Cell Biol. 78, 91–102 (2000). 10762408 10.1046/j.1440-1711.2000.00888.x PMC7159383

[b42] Gill M. A. The role of dendritic cells in asthma. J. Allergy Clin. Immunol. 129, 889–901 (2012). 22464668 10.1016/j.jaci.2012.02.028

[b43] Condon T. V., Sawyer R. T., Fenton M. J. & Riches D. W. H. Lung dendritic cells at the innate-adaptive immune interface. J. Leukoc. Biol. 90, 883–895 (2011). 21807741 10.1189/jlb.0311134 PMC3206474

[b44] Hammad H. & Lambrecht B. N. Dendritic cells and epithelial cells: linking innate and adaptive immunity in asthma. Nat. Rev. Immunol. 8, 193–204 (2008). 18301423 10.1038/nri2275

[b45] Möller G. M. . Increased numbers of dendritic cells in the bronchial mucosa of atopic asthmatic patients: Downregulation by inhaled corticosteroids. Clin. Exp. Allergy 26, 517–524 (1996). 8735863

[b46] Divekar R. & Kita H. Recent advances in epithelium-derived cytokines (IL-33, IL-25, and thymic stromal lymphopoietin) and allergic inflammation. Curr. Opin. Allergy Clin. Immunol. 15, 98–103 (2015). 25479313 10.1097/ACI.0000000000000133 PMC4346181

[b47] Ziegler S. F. The role of thymic stromal lymphopoietin (TSLP) in allergic disorders. Curr. Opin. Immunol. 22, 795–799 (2010). 21109412 10.1016/j.coi.2010.10.020 PMC3032269

[b48] Smith D. E. IL-33: a tissue derived cytokine pathway involved in allergic inflammation and asthma. Clin. Exp. Allergy 40, 200–208 (2010). 19906013 10.1111/j.1365-2222.2009.03384.x

[b49] Saluja R., Khan M., Church M. K. & Maurer M. The role of IL-33 and mast cells in allergy and inflammation. Clin. Transl. Allergy 5, 33 (2015). 26425339 10.1186/s13601-015-0076-5 PMC4588911

[b50] Moussion C., Ortega N. & Girard J.-P. The IL-1-Like Cytokine IL-33 Is Constitutively Expressed in the Nucleus of Endothelial Cells and Epithelial Cells *In Vivo*: A Novel ‘Alarmin’? PLoS One 3, e3331 (2008). 18836528 10.1371/journal.pone.0003331 PMC2556082

[b51] Snelgrove R. J. . Alternaria-derived serine protease activity drives IL-33–mediated asthma exacerbations. J. Allergy Clin. Immunol. 134, 583–592.e6 (2014). 24636086 10.1016/j.jaci.2014.02.002 PMC4152000

[b52] Nakanishi W. . IL-33, but Not IL-25, Is Crucial for the Development of House Dust Mite Antigen-Induced Allergic Rhinitis. PLoS One 8, e78099 (2013). 24205109 10.1371/journal.pone.0078099 PMC3808342

[b53] Kamekura R. . The role of IL-33 and its receptor ST2 in human nasal epithelium with allergic rhinitis. Clin. Exp. Allergy 42, 218–228 (2012). 22233535 10.1111/j.1365-2222.2011.03867.x

[b54] Cayrol C. & Girard J.-P. IL-33: an alarmin cytokine with crucial roles in innate immunity, inflammation and allergy. Curr. Opin. Immunol. 31, 31–37 (2014). 25278425 10.1016/j.coi.2014.09.004

[b55] Liu Y.-J. . TSLP: An Epithelial Cell Cytokine that Regulates T Cell Differentiation by Conditioning Dendritic Cell Maturation. Annu. Rev. Immunol. 25, 193–219 (2007). 17129180 10.1146/annurev.immunol.25.022106.141718

[b56] Ying S. . Thymic stromal lymphopoietin expression is increased in asthmatic airways and correlates with expression of Th2-attracting chemokines and disease severity. J. Immunol. 174, 8183–90 (2005). 15944327 10.4049/jimmunol.174.12.8183

[b57] Ito T. . TSLP-activated dendritic cells induce an inflammatory T helper type 2 cell response through OX40 ligand. J. Exp. Med. 202, 1213–1223 (2005). 16275760 10.1084/jem.20051135 PMC2213234

[b58] Bordon Y. Allergy: Crystal clear culprit. Nat. Rev. Immunol. 11, 304–304 (2011). 10.1038/nri2985 21508979

[b59] Kool M. . An Unexpected Role for Uric Acid as an Inducer of T Helper 2 Cell Immunity to Inhaled Antigens and Inflammatory Mediator of Allergic Asthma. Immunity 34, 527–540 (2011). 21474346 10.1016/j.immuni.2011.03.015

[b60] Kong J. . Comprehensive metabolomics identifies the alarmin uric acid as a critical signal for the induction of peanut allergy. Allergy 70, 495–505 (2015). 25647422 10.1111/all.12579

[b61] Hara K. . Airway Uric Acid Is a Sensor of Inhaled Protease Allergens and Initiates Type 2 Immune Responses in Respiratory Mucosa. J. Immunol. 192, 4032–4042 (2014). 24663677 10.4049/jimmunol.1400110 PMC4013745

[b62] Idzko M. . Extracellular ATP triggers and maintains asthmatic airway inflammation by activating dendritic cells. Nat. Med. 13, 913–919 (2007). 17632526 10.1038/nm1617

[b63] Jairaman A., Maguire C. H., Schleimer R. P. & Prakriya M. Allergens stimulate store-operated calcium entry and cytokine production in airway epithelial cells. Sci. Rep. 6, 32311 (2016). 27604412 10.1038/srep32311 PMC5015156

[b64] Sudha V. T., Arora N., Gaur S. N., Pasha S. & Singh B. P. Identification of a serine protease as a major allergen (Per a 10) of Periplaneta americana. Allergy Eur. J. Allergy Clin. Immunol. 63, 768–776 (2008). 10.1111/j.1398-9995.2007.01602.x 18445191

[b65] Goel C. . Serine protease Per a 10 from Periplaneta americana bias dendritic cells towards type 2 by upregulating CD86 and low IL-12 secretions. Clin. Exp. Allergy 42, 412–422 (2012). 22356142 10.1111/j.1365-2222.2011.03937.x

